# Human movement patterns predict task-unrelated thought

**DOI:** 10.1371/journal.pone.0341902

**Published:** 2026-01-28

**Authors:** Aaron Y. Wong, Anaëlle E. Charles, Caitlin Mills, Nick Stergiou, Aaron D. Likens

**Affiliations:** 1 Department of Educational Psychology, University of Minnesota, Minneapolis, Minnesota, United States of America; 2 Division of Biomechanics and Research Development, Department of Biomechanics, Center for Research in Human Movement Variability, University of Nebraska at Omaha, Omaha, Nebraska, United States of America; 3 Division of Sleep Medicine, Harvard Medical School, Boston, Massachusetts, United States of America; 4 Department of Anesthesia, Critical Care, and Pain Medicine, Massachusetts General Hospital, Boston, Massachusetts, United States of America; 5 Department of Physical Education and Sport Science, AUTH Biomechanics, Aristotle University of Thessaloniki, Thessaloniki, Greece; Leuphana University of Lüneburg: Leuphana Universitat Luneburg, GERMANY

## Abstract

The cognitive phenomenon, known as task-unrelated thought, reflects the attention shift of one’s mind away from the task at hand. Evidence suggests that task-unrelated thought occurs in 30–50% of people’s waking time. Previous research using the metronome response task shows that task-unrelated thought is related to variability in response time magnitude. However, those studies did not account for the time varying characteristics of an individual’s tapping behavior. In the current study, three research questions were investigated: (1) What is the relationship between task-unrelated thought and movement dynamics (finger tapping dynamics)? (2) How does the statistical structure of external stimuli influence task-unrelated thought? (3) Does this structure moderate the relationship between task-unrelated thought and finger tapping dynamics? Participants performed the metronome response task under four different metronome structures: NoTone, Persistent, Periodic, and Random. Participants synchronized their finger to the metronome tone for each condition and self-reported the occurrence of task-unrelated thought. Overall, an increase of the Hurst exponent resulted in a decrease of task-unrelated thought probability. The findings have implications that behavioral variability has value in detecting task-unrelated thought. Additionally, studies using the metronome response task should account for the impact of the tone structure being used. Future research is warranted in this field to truly understand the mechanism behind task-unrelated thought and its link to human movement variability.

## Introduction

It is common for people to dissociate their thoughts from their ongoing tasks, even though to an observer they may seem to be fully engaged. This phenomenon is referred to as task-unrelated thought (TUT), also commonly known as “mind-wandering” [1]. TUT occurs when thoughts drift away from the task at hand and is estimated to occur nearly 30–50% of the time we are awake [2–4]. To date, research suggests that TUT plays an important role in many facets of our lives, having either a positive or negative impact [5–9]. On one hand, TUT has been shown to contribute to solving complex problems (i.e., insightful thought can lead to “eureka” moments) [5]. On the other, TUT has also been related with a decrease in cognitive and motor performance across a variety of tasks, presented as slower reaction times, lower scores in task accuracy, and decreased reading comprehension [1,7,8,10–14]. However, our understanding of how TUT affects motor task performance, specifically, is limited. Of particular interest is how subtle, variable patterns in movement sequences map onto the wandering mind.

Repetition is a fundamental feature for any number of human behaviors, such as gait cycles [15–20], finger taps [21–22], reaction times [23], and time estimation [24]. Repetitions of these actions have an average duration, but small temporal fluctuations are present from one action to the next, such that two consecutive actions are never identical. Such fluctuations are not limited to intentional actions [16,25–27]. An example can be seen in human heartbeats which are assumed by many laypeople as being somewhat regular, with a healthy resting heart rate around 60 beats per minute; however, heart rate tends to vary considerably throughout the day [28–30]. Across both behavioral and physiological domains, fluctuations are thought to reveal important information about the health and adaptability of the system that generates them [19,20,31]. Because TUT also displays a pattern of fluctuating repetition, as it varies intermittently from one moment to the next and from an attentive state to an inattentive state [12,32], a question is raised: can behavioral fluctuations also reveal whether our mind has drifted off-task? In other words, are our cognitive and motor systems interconnected in the sense that failure in one leads to failure in another.

Research work using the metronome response task suggested that TUT could be related to movement variability [12,33–36]. In this task, participants listened to a steady beat (metronome) and were asked to make a key response whenever a tone was heard. Participants were probed for TUT periodically in between tapping intervals. The key metric regarding movement was the amount of variability in finger tapping, which was simply assessed via the standard deviation of the response times relative to the tones. In the previous studies, increased rates of TUT were associated with increased amounts of variability in response to the tone [12,36]. According to the executive failure hypothesis, the variation in response times may represent failures in the allocation of executive control resources to the task (i.e., TUT), thus disrupting optimal motor responses [6,32]. However, a potential limitation of these previous studies was that variability was measured in terms of standard deviation. Standard deviation is a basic summary statistic that does not inherently capture the time-varying relationship among subsequent actions; the standard deviation simply represents an average of distances of the distribution values from the mean, thus estimating the *amount of variability*. However, research has shown that the rate of TUT can change due to temporal factors [37], and therefore it may not capture the whole picture. To capture the fluctuations that occur over time and across multiple repetitions of a task, or the *temporal structure of variability*, a different class of metrics is needed [19]. One such group of metrics is referred to as *fractal analysis* and has been widely deployed to study fluctuations in numerous physiological and movement contexts [17,28,30]. Fractal analysis has also been used in previous work to distinguish subtle temporal changes in healthy behavior from pathological patterns in a variety of situations when linear metrics such as standard deviations have shown to be inadequate [20,28,30,38]. The current study thus leverages fractal analysis to investigate if there is a link between movement variability (structure) and TUT by examining the temporal relationships between movement dynamics and instances when a person’s thoughts drift off-task.

**Fig 1:**
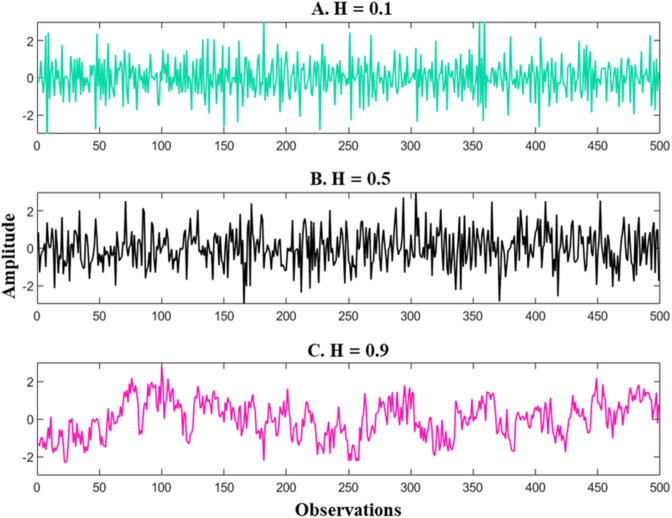
Example time series with different Hurst exponents. Note: The time series have the same mean and standard deviation but different temporal structures. Panel (a) is an antipersistent time series with H = 0.1, while panels (b) and (c) reflect random and persistent processes with H = 0.5 and H = 0.9, respectively.

One way to implement fractal analysis is through the examination of long-range correlations (LRC), which implies that a behavior observed at one point in time relates to many successive repetitions of that same behavior. For example, fractal analysis has been used to identify if neurophysiological and behavioral time series exhibit the presence of LRC (i.e., heart rate, neural activity, human walking) [16,23,28,39–44]. LRC can be captured by what is known as the Hurst exponent (H), a parameter referring to the tendency that an event in a time series is followed by a similar event [30,43,45,46]. Fig 1 depicts several time series with different H exponents. Note that all the time series have the same mean and standard deviation but differ in terms of their temporal structure. When H = 0.5, the observations are not correlated, meaning they are independent from one point in time to the next (Fig 1B). Deviations from H = 0.5 can either tend toward 0 or 1. In the case where H ranges from 0 to 0.5, the time series is characterized as *anti-persistent* (Fig 1A); and when H ranges from 0.5 to 1, the time series is referred to as *persistent* (Fig 1C). Anti-persistent simply means that successive values are statistically more likely to be followed by values of the opposite sign, while persistent means that successive values are statistically more likely to be followed by values of the same sign. Ubiquitous in all biological systems, the presence of persistent values is thought to be a prime characteristic of healthy and adaptive behavior [30,31,44,47]. As such, any differentiation from this type of a temporal structure can potentially be used as a marker to detect abnormality in the system.

There are many reasons to believe that persistent values (0.5 < H < 1) in motor behavior may be related to TUT. This argument is based on recent literature from several disciplines and medical areas, including brain function and disease dynamics, which have shown that many apparently “noisy” phenomena are the result of nonlinear interactions and have deterministic origins [48–56]. Based on this information, the optimal movement variability hypothesis (OMVH) was proposed which posits that the natural fluctuations that are present in normal motor tasks (i.e., the temporal structure of variability) are characterized by an appropriate or optimal state of variability [20,31,47]. Optimal variability is associated with complex interactions across multiple control systems, feedback loops, and regulatory processes that enable an organism to function and adapt to the demands of everyday life [20,31,47]. This physiological complexity is recognized as an inherent attribute of healthy biological systems, whereas the loss of complexity with aging and disease is thought to reduce the adaptive capabilities of the individual [15,25,28]. A loss of complexity can refer to either an overly constrained, periodic system, or an overly random, incoherent system. Complexity can be estimated by examining the H exponent of the fluctuations that are present in the task under investigation linking it to persistence [57–58]. Optimal movement patterns typically produce H close to 1. In contrast, deviation away from the optimal movement patterns tend towards 0.5. In support of this hypothesis, research has shown that healthy motor behavior presents such values [17,22,33,49]. Other studies showed that people recover faster from mechanical disturbances when they walk with stride-to-stride fluctuations that exhibit values of H closer to 1 [59–60]. Moreover, evidence strongly suggests that similar values of H are present in the interface between healthy human cognition and physiological processes, reflecting a possible interaction between mind and body [40,61–72]. For example, such values have been found in upper limb movements as related to task engagement in tasks like driving [73]. These findings suggested that there could be a connection between the temporal relationships of movement dynamics and instances when a person’s thoughts drift off-task. Possibly, if a decrease in task performance, such as a slower reaction time or a decrease in task accuracy has been related with TUT [8,74,75], then we can hypothesize that deviations from optimal movement patterns would likewise suggest a lack of attention due to TUT. This would suggest that the two systems are interconnected and could have implications for how the occurrence of TUT is related to other physiological processes.

In addition to the overall relationship between TUT and movement variability, we expect that TUT is likely to be task sensitive. This prediction builds from previous work suggesting that the frequency of TUT tends to be modulated by task difficulty, as more attentional control is required during challenging tasks, thus “allowing” for less TUT to occur [6,32,76]. This finding has implications for tasks in which we want to study human movement variability while our minds also have the potential to wander, e.g., walking. In prior studies that used the metronome response task, the same metronome frequency was used across all participants [12,33]. Thus, the individual’s natural variability patterns were not considered in the development of the metronome, i.e., the difference between the metronome and a participant’s natural tapping pattern would affect how much executive control is required. Here, we also wanted to investigate how this potential limitation may have affected previous results. We thus developed an experimental paradigm, inspired by studies in gait variability, that takes into account the effect of the temporal structure of the metronomic apparatus on the frequency of TUT [21,66,77–80]. In our experimental paradigm and according to the OMVH, natural tapping patterns should be disturbed by classic metronomes with isochronous, periodic inter-beat time intervals or variable metronomes that exhibit random inter-beat time intervals. From this perspective, tapping to such “unnatural” metronomes might be perceived as more difficult to sustain, requiring more attentional control, and allowing less TUT to occur.

Therefore, in the present study we raised three research questions: (1) What is the relationship between TUT and movement dynamics in terms of finger tapping dynamics? (2) How does the temporal structure of the variability in the external stimuli influence TUT? (3) Does the temporal structure of the variability in the external stimuli modulate the relationship between TUT and finger tapping dynamics? To answer these questions, participants performed the metronome response task under four different conditions: *NoTone, Periodic, Persistent,* and *Random*. In the *NoTone* condition, participants tapped their finger at their own pace. For the other conditions, the frequency content of a metronome was calibrated to the participant’s natural tapping frequency with temporal fluctuations implemented in the inter-beat intervals based on certain time series. Essentially, participants were instructed to synchronize their tapping to the metronome beats in which each condition differed in terms of its temporal structure. For the *Periodic* condition, participants synchronized their tapping to an isochronous or invariant beat time series. For the *Persistent* condition, participants synchronized their tapping to a variable and structured beat time series that exhibited high complexity as defined with an H close to 1. For the *Random* condition, participants synchronized their tapping to a random (non-correlated) beat time series as defined with an H equal to 0.5. Participants also self-reported TUT instances to examine how TUT’s occurrence relates to the different metronome structures.

Based on the executive failure hypothesis, we hypothesized that TUT will be more likely to occur when tapping behavior is closer to a participant’s natural tapping pattern due to less need for executive control to maintain the tapping behavior. Additionally, we hypothesize that TUT will be more likely to occur when tapping in the *NoTone* condition (the natural frequency) and/or the *Persistent* condition in which tapping occurs to a stimulus that exhibits high complexity. That is because in those conditions, participants will be tapping in either their natural frequency pattern or one that is preferred due to its high complexity. Therefore, less executive control will be needed to perform the task increasing the likelihood of TUT. In contrast, we also hypothesized that TUT will be less likely to occur when the temporal structure of the metronome is either periodic or random. The rationale is that in those conditions, participants will have to adapt their tapping pattern to unnatural patterns. Thus, more executive control will be needed to perform the task, reducing the likelihood of TUT. Lastly, we hypothesized that we would observe an effect of the metronome condition on the relationship between TUT and finger tapping dynamics. More specifically, when synchronizing motor behavior to an external stimulus, individuals tend to exhibit the same temporal structure that was embedded in the given stimuli [66,77,81,82]. Therefore, we hypothesized that the temporal structure of the metronomes will simultaneously influence finger tapping and the probability of TUT.

## Methods

### Participants

One hundred and twenty undergraduates with normal and assisted hearing were recruited from the University of New Hampshire. All participants completed an informed, written consent electronically and were compensated with course credit. The study was approved by the Institutional Review Board at the University of New Hampshire and determined to be exempt in accordance with relevant federal guidelines.

### Procedure

The experiment was conducted online with a single within-subjects manipulation. Upon signing the informed consent, participants completed a finger tapping task to determine participants’ baseline tapping rate. Participants were instructed to press the letter “M” at a self-selected pace that they could easily maintain. The finger tapping task lasted for 1 minute. The mean and standard deviation of finger tapping rate was calculated and used to calibrate the metronome pace in the metronome conditions for each participant. Participants then completed a sound check in which a sample tone was played to allow participants to adjust the sound of their audio to a comfortable level.

Next, participants performed the metronome response task under four conditions each lasting 8 minutes: three metronome conditions, *Periodic*, *Persistent*, and *Random,* and one without a metronome, *NoTone*. For the metronome conditions, participants were instructed to synchronize their finger taps to the given metronome by pressing the letter “M’‘ whenever they heard a tone. For the *Periodic* condition, participants synchronized their taps to an invariant metronome (i.e., traditional metronome). For the *Persistent* condition, participants synchronized their tapping to a variable and temporally structured metronome, a tone’s time series exhibiting a positive correlation (H = 1.0). For the *Random* condition, participants synchronized their tapping to a non-correlated series of tones (H = 0.5). The Hurst exponents for the Persistent and Random condition have been used in prior research synchronizing motor movements with different metronome structures [59, 66, 81-82]. Lastly, during the *NoTone* condition participants did not hear a metronome and were instructed to press the letter “M” at a self-selected pace, similar to the initial finger tapping baseline task. The order of the conditions was counterbalanced using a Latin square design.

**Fig 2:**
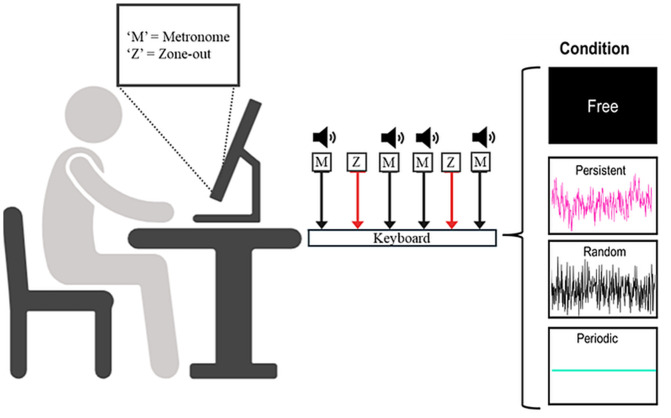
Metronome response task setup. Note: Participants completed the experiment on their personal computer. While participants did the task, keypress instructions were displayed on the screen. Participants pressed ‘M’ in response to a tone and ‘Z’ whenever they found themselves having task-unrelated thoughts. Participants completed the task under all four metronome conditions.

For all the conditions, participants were also instructed to press the letter “Z” on the same keyboard every time they experienced having any task-unrelated thoughts (e.g., plans for the weekend, a discussion with a friend or family, etc.) (See Fig 2). This self-caught method for assessing TUT is common in the literature and has been found to be a reliable way to assess TUT [83]. Upon completing the task, participants completed a demographic survey. The duration of the entire experiment lasted 35–45 minutes.

### Time series and statistical analysis

The three metronomes used in the metronome response task were created and calibrated based on each participant’s baseline tapping rate. The *Periodic* metronome was scaled using the participant’s inter-taps mean with a standard deviation of zero. The *Persistent* and *Random* tones were scaled from a preconstructed, normalized base structure as has been done in prior studies [59]. For the *Persistent* condition (H = 1.0), the metronome was scaled using the inter-taps mean and standard deviation of the participant’s baseline taps. Finally, for the *Random* condition (H = 0.5), the metronome was scaled based on the participant’s inter-taps mean and a random standard deviation generated using a Gaussian normal distribution.

As a compliance check, the number of taps was compared to the expected number of taps (number of tones) to ensure that the metronome response task was performed correctly. For each condition with a metronome, the tapping trial was considered to be valid if the number of taps was within 10% of the expected taps. If the number of taps fell outside the 10% confidence interval, the trial was excluded for statistical analysis. Based on the exclusion criterion, 66 *Periodic*, 64 *Persistent*, and 67 *Random* trials were included in the statistical analysis. Trials in the *NoTone* condition were not held to the same criterion as participants may naturally deviate from their initial tapping behavior without a metronome to keep the same pace. However, 29 trials were excluded due to not performing the task correctly (e.g., long periods of time without a response). After applying the exclusion criteria, a total of 66 *Periodic*, 64 *Persistent*, 67 *Random*, and 91 *NoTone* trials were included in the analyses.

The first 455 seconds (out of the total 480 seconds) of each trial was divided into 7 periods of 65 second intervals. Prior research using the Metronome Response Task has shown that this is a sufficient time window for TUT to occur [36,84]. Traditionally, Detrended Fluctuations Analysis (DFA) has been the gold standard to measure statistical persistence in human movement and cognitive sciences [32,85]. However, DFA has some limitations. Best practices suggest that DFA requires at least 500 points for the results to be reliable [42,48,86,87]. In this study the tapping time series was inferior to 500 points, therefore to overcome this limitation, we used the Bayesian approach by [85] to compute the Hurst-Kolmogorov Process (HKprocess). Commonly used in other research areas, the HKprocess is a posterior distribution of the ϕ parameter of the autoregression process using a Bayesian approach and has shown reliable performance on shorter time series (n < 500) [88–91]. Upon sectioning the time series in 7 periods, the temporal structure of individuals’ tapping performance was evaluated within each period.

To answer the research questions, statistical analyses were performed with logistic linear mixed effects models using template model builder from the glmmTMB package in R [88]. The dependent variable, TUT, was treated as a binary variable with occurrence depending on if any TUT event was reported within a time period (presence of TUT = 1; absence of TUT = 0). The Hurst exponent of tapping behavior was treated as a continuous fixed effect and was z-scored across participants. For analyses that used metronome condition as a moderator, metronome condition was treated as a discrete fixed effect. For comparisons between conditions, the *NoTone* condition was treated as the reference condition. Although no predictions about the effects of time were made, linear and quadratic terms for period number were also included as continuous covariates (fixed effects) to control for time effects—prior research has shown that TUT likelihood increases along with task duration [14]. Random intercepts for participant and random slopes for metronome condition within participant were also included.

## Results

### What is the effect of metronome condition on movement dynamics (finger-tapping dynamic)?

As a manipulation check, we first investigated the effect of the metronome condition on the tapping behavior as this pattern should align with previous studies (see Table 1). A linear mixed effect model was performed with Hurst as the dependent variable, metronome condition as a discrete, fixed effect, and participant as a random effect. *NoTone* had a lower H than *Persistent*, β = −.16, SE = .05, p = .02, but had a higher H than *Random*, β = .56, SE = .05, p < .001, and *Periodic*, β = 1.20, SE = .05, p < .001. *Persistent* had a higher H than *Random*, β = .72, SE = .06, p < .001, and *Periodic*, β = 1.36, SE = .06, p < .001. *Random* had a higher H than *Periodic*, β = 0.64, SE = .05, p < .001. Although the H exponents are, on average, slightly lower than those often observed in previous work [77–78] the general trends supported by significant differences among conditions suggest that the metronome manipulation was successful.

**Table 1:** Average Hurst exponent and TUT binary responses by condition.

	H		TUT	
Condition	*M*	*SD*	*M*	*SD*
NoTone	0.58	0.14	0.64	0.48
Persistent	0.61	0.15	0.73	0.44
Periodic	0.36	0.19	0.68	0.47
Random	0.48	0.14	0.68	0.47

Note: Hurst exponent ranges from 0 to 1 and TUT probability ranges from 0 to 1.

### Main analysis

In order to examine the three research questions, the model contained linear and quadratic effects of time, metronome condition, H, and the interaction between metronome condition and H. All reported results are based on the final model. Though it was not key to the experiment, we first note that period number was predictive of TUT, with both the linear and quadratic terms being related. More specifically, the linear term suggested that a one-unit change in period number is expected to increase the log-odds of a TUT event by 0.45 (*β* = 0.45, SE = 0.16, *p* = .004). The quadratic term suggests a concave-down curvilinear change in the log-odds of TUT (*β* = −0.06, SE = 0.02, *p* = .004). This finding is consistent with prior research demonstrating a general trend of TUT over time [14].

### What is the relationship between TUT and movement dynamics (finger-tapping dynamics)?

**Fig 3:**
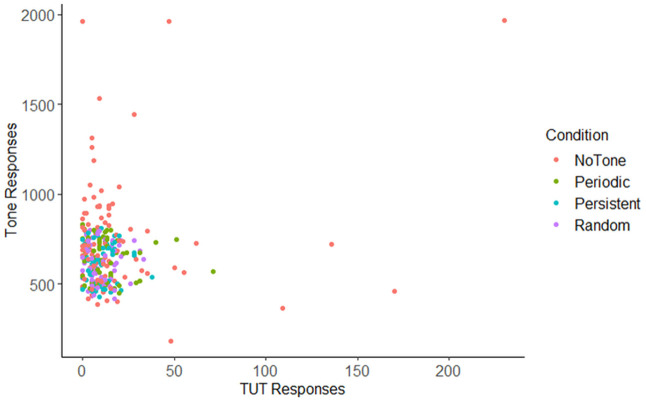
TUT responses and finger tapping responses by condition.

Fig 3 shows TUT responses and finger-tapping responses by metronome condition. Early model building steps suggested a one SD increase in H of tapping behavior is expected to decrease the log-odds of a TUT event by 0.39 (β = −0.39, SE = 0.17, p = .023). In other words, deviations away from the optimal form of variability decrease the tendency for TUT.

### How does the temporal structure of the external stimuli influence TUT?

Table 1 shows TUT rates by metronome condition. Results suggested that, on average, the *Persistent* condition increased the log-odds of TUT events by 0.75 when compared to the *NoTone* condition (*β* = 0.75, SE = 0.34, *p* = .028). However, TUT likelihood did not significantly differ between the *NoTone* and *Random* conditions (*β* = −0.01, SE = 0.27, *p* = .963), nor between the *NoTone* and *Periodic* condition (*β* = 0.06, SE = 0.31, *p* = .850).

### Does the temporal structure of external stimuli moderate the relationship between TUT and finger-tapping dynamics?

**Fig 4:**
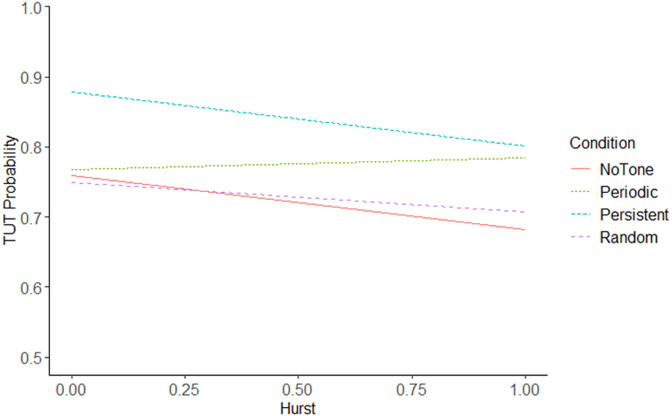
TUT probability as a function of the Hurst exponent by condition. Note: Regression lines were obtained from the linear mixed effect model.

The interaction between the structure of tapping behavior (H) and the metronome conditions was examined to determine if the temporal structure of the metronome tone was a moderator of the relationship between metronome condition and TUT. Simple slopes analyses were performed to examine the interaction. Fig 4 shows the relationship between TUT and H by condition. The *NoTone* condition did not differ in the relationship between H and TUT with the *Periodic* condition, *β* = −0.49, SE = 0.23, *p* = .147, the *Persistent* condition, *β* = 0.09, SE = 0.28, *p* = .990, or the *Random* condition, *β* = −0.13, SE = 0.27, *p* = .961. The relationship between H and TUT did not differ between the *Periodic* condition and the *Persistent* condition, *β* = 0.57, SE = 0.27, *p* = .153, or the *Random* condition, *β* = 0.35, SE = 0.26, *p* = .530. No difference was observed between the *Persistent* condition and the *Random* condition, *β* = −0.22, SE = 0.30, *p* = .879. Though no comparisons were significant, the *Periodic* condition had a positive relationship between H and TUT (*β* = 0.09, SE = 0.15), while the Persistent, (*β* = −0.48, SE = 0.22), Random, (*β* = −0.26, SE = 0.21), and NoTone, (*β* = −0.39, SE = 0.15), conditions had a negative relationship.

### Additional analyses

The current study examined the relationship between the structure of variability and TUT while prior studies have used the magnitude of variability. Because both are measures of variability, it is possible that the structure of variability is a more calculation-intensive version of the magnitude of variability. In this case, it would be simpler to use magnitude of variability instead. An additional analysis was run to examine if the two measurements independently predict TUT. Because of how the metronomes were constructed in the current study, the magnitude of variability could not be calculated in the same way as previous studies. Prior research using the metronome response task have used consistent inter-tone intervals of 1300 ms. In the current study, the average baseline tapping rate was ~ 850 ms and the interval would vary in the Persistent and Random metronomes. Therefore, participants could fail to respond to a tone or make multiple responses to a tone making it difficult to determine a 1–1 mapping of taps to tones which is required to calculate prior studies’ magnitude of variability. As an alternative, magnitude of variability was calculated as the standard deviation of the inter tap intervals.

Magnitude of variability was added to the main model as a fixed effect and allowed to interact with metronome condition and H. Magnitude of variability was not predictive of TUT, (*β* = 0.01, SE = 0.01, *p* = .486), and no interactions involving magnitude of variability were significant. Additionally, all previous significant results were still significant. This suggests that the structure of variability is a different construct than the magnitude of variability and is related to TUT differently.

## Discussion

In the current study, the metronome response task was used to investigate the relationship between the temporal structure of finger-tapping and TUT. We performed a within-subjects experimental design in which participants were asked to synchronize their finger taps to three metronome conditions: *Periodic*, *Persistent*, and *Random*. In a fourth condition (*NoTone*), participants performed a self-selected pace tapping condition (no metronome). A self-caught method was used to assess TUT during all conditions. Unlike previous studies focusing on finger-tapping variability (standard deviations) within the tapping task [12,33,35], the current study investigated the nature of the relationship between the temporal structure of finger-tapping and TUT events using fractal analysis. The current study aimed to address the following questions: (1) What is the relationship between TUT and movement dynamics in terms of finger tapping dynamics? (2) How does the temporal structure of the variability in the external stimuli influence TUT? (3) Does the temporal structure of the variability in the external stimuli modulate the relationship between TUT and finger tapping dynamics? Answering those questions requires a matter of care because the picture painted from our modeling results suggest that the answers are not independent of one another. In this discussion, we synthesize answers to those questions that acknowledge that interdependency.

### Tapping variability and metronome structure jointly influence TUT

Previous research found that TUT increases with increasing magnitude of tapping variability [12,33,35]. Our main goal in this study was to complete the story by considering a second aspect of variability—its structure—as it relates to TUT. As a general note, our results echo those from the larger motor control literature showing that unconstrained tapping variability produces patterns consistent with long range correlations, as seen in gait as well as other cognitive behaviors [24,41,63,64,66,70,74,78,81,82,89]. Furthermore, our manipulation check supported the idea that tapping variability is also amenable to experimental manipulation with metronomes. Indeed, the *Periodic* and *Random* conditions weakened or eliminated tap to tap correlations, as expected. Given that our manipulations were successful in modifying tapping variability, the main lingering issues concern how those manipulations influenced TUT.

Overall, there was a negative relationship between H and TUT. This was observed in three conditions (*NoTone*, *Persistent,* and *Random*), as increasing tapping structure (i.e., increasing H) tended to reduce TUT. In those conditions, participants were less likely to experience TUT when tapping behavior was more like patterns observed in unconstrained repetitive movements [30,31,39,43,44,47]. However, in the *Periodic* metronome an increase in tapping structure was associated with a decrease in TUT, that effect clearly depended on the resultant H derived from the tapping series. Though the interaction between the structure of tapping behavior (H) and tapping conditions on TUT was not significant, the trends suggest *Periodic* metronomes could potentially affect behavior differently and is worth future exploration. Disentangling that difference between the *Periodic* and all other conditions may benefit from consideration of the gait literature that has used similar metronomes.

Two recent studies investigated how gait is influenced by two components of variability, the autocorrelation (as in the present study) and the probability distribution function (PDF) of the inter-beat intervals [66,89]. Specifically, the H of the metronome was manipulated to be either 0.5 (random metronome) or 1 (persistent). As predicted, gait paced by a persistent metronome produced persistent inter-stride intervals that were essentially indistinguishable from unpaced walking. Furthermore, gait paced by a random metronome tended to become random itself. Additionally, metronomes constructed to have a uniform PDF were more disruptive when compared to metronomes constructed to have a normal (Gaussian) distribution. This is relevant because unpaced inter-stride intervals tend to be normally distributed with a persistent structure, and metronomes that deviated the most from typical unpaced walking patterns exerted the greatest influence on gait. The results from those studies, and others like them, seem to imply that the sensorimotor system has an affinity for normally distributed variability with a persistent structure.

In the present study, although the *Persistent* condition tended to increase TUT relative to the *NoTone* condition, the H-TUT relationship was similarly negative across all conditions except the *Periodic* condition. In that sense, there seems to be a parallel between the present results and the gait results reported in the previous paragraph [66,89]. Although their autocorrelation functions clearly differ, the *Random* and the *Persistent* conditions have in common a normal distribution. That means that, in some sense, the *Periodic* condition, with its unchanging interbeat intervals, differs most from the other conditions. Hence, the flattening of the H-TUT relationship in that condition could be explained on similar grounds. The disruption to the H-TUT relationship could result from the *Periodic* condition’s greater deviation from patterns of variability for which the sensorimotor system has a stronger affinity, as evident from the distinct H*-*TUT relationship in that condition. However, additional research is needed to test this explanation.

### The role of tapping variability in TUT

The present findings complement previous results suggesting that an increase in behavioral variability tends to be associated with an increase in TUT [33–36,92]. Recall that the distinction between those studies and the present work is that those studies focused on magnitude of variability and its relationship to TUT, whereas the current study is concerned with the implication the structure of variability has for TUT. Although the approaches to variability are distinct, when considered together, the pattern of results across methodologies has an analogue in the motor control literature [39,90].

As noted in the introduction, the historical and even contemporary motor control literature often refers to variability as originating from error or noise [27,90]. As such, variability in human movements such as gait were first understood from that perspective and provided a wealth of information about how the magnitude of variability changes as a function of practice and learning [90,93,94]. Later studies also showed that the magnitude of variability also surfaces as an important indicator of motor problems that emerge due to neurodegenerative diseases and the natural course of aging [95–99]. The general pattern of results is that variability tends to decrease as we hone skills through learning and practice [23,39,97,99,100]. Moreover, older adults and those with neurological and physical impairments tend to be more variable than their younger, healthier counterparts [96–98,101]. Thus, there seems to be an inverse relationship between those trends and the ones discussed in the introduction concerning the structure of variability and its relationship to health and disease. While magnitude of variability is greater for novices and those with sensorimotor problems, the opposite is true for structure, with experts and healthy young adults producing the most structured variability [20,42,47,50,81,82,102,103].

Those comparisons parallel the current findings when considered in the context of the larger literature [12,33,34]. Increases in TUT seem to be accompanied by increases in the magnitude of variability while the structure of variability decreases. What emerges is a view with two types of variability, both important for understanding human behavior [104]. From the magnitude perspective, as attention to task waxes and wanes throughout a task’s duration, more frequent and potentially longer bouts of TUT emerge [14,32,34,92]. Indeed, our modeling results show that TUT tends to increase over time, even after accounting for the effects of tapping condition. The consequence is an ever-broadening distribution of inter-tap intervals. From the structural perspective, at the heart of this work, TUT seems to decrease as the structure of variability tends towards its theoretical maximum (i.e., H = 1) but increases as that temporal structure degrades. The ebb and flow of task-related and task-unrelated thought leads to one of two potential outcomes in terms of temporal structure. Fleeting attention could lead to a completely incoherent sequence of interbeat intervals as the participant pecks randomly from one tap to the next. Intermittent attention could also lead to an antipersistent regime, which is often interpreted as evidence of a corrective process [105–107]. Both of those possibilities were realized in our experimental data.

The current results, accompanied by the larger literature, suggest that both the magnitude and the structure of variability hold value in detecting TUT but reveal different characteristics. Measures of magnitude that are clearly important are, by definition, unable to resolve changes in temporal structure that may likewise be strong indicators of a wandering mind. The addition of time series methods designed to capture such changes enhances the value of tapping variability in assessing TUT.

### The structure of stimuli in tapping paradigms

On a more methodological note, the current results suggest that selection of cues in tapping paradigms is not a trivial matter. Our results clearly demonstrate that the structure of the underlying metronome influences the structure of tapping time series while simultaneously influencing TUT. The *Persistent* condition increased the likelihood of TUT relative to the *NoTone* condition and across most conditions, H decreased as TUT increased, although that was not always the case. The finding showing that the H-TUT behavior differs in the *Periodic* condition relative to the others has important implications, because prior studies have only used a standard (*Periodic*) metronome which could be an issue of generalizability to a person’s everyday behavior [12–33].

### Potential limitations

There are some limitations to the current study. First, because of the variable timing in between tones, we were unable to determine how well participants followed the metronome tones and consequently magnitude of variability. Though only trials within 10% of the expected taps were used in the analyses, the possibility that a participant was not following the metronome structure remains. Future research should examine how the magnitude and structure of variability independently affect TUT.

Second, the possibility exists that the tapping behavior during the initial baseline period and *NoTone* condition were different (different means and standard deviations for the interval between taps). In our study, the *Persistent*, *Periodic*, and *Random* conditions were constructed based on tapping behavior during the baseline period. Previous research has shown that human movement variability (structure) changes over time [25,29,30,69], that is, it is possible that the tapping behavior displayed during the 1-minute baseline period might differ from the tapping behavior during the 8 minutes in the *NoTone* condition. Therefore, the *Persistent*, *Periodic*, and *Random* conditions may not actually be based on a participant’s natural tapping behavior.

Third, the current study used self-caught measures for TUT. Another common method for capturing TUT is using probes [108]. The current study used self-caught measures because probes would interfere with the metronome structure by introducing pauses in order to collect responses. Unlike probe-caught, self-caught measures require meta-awareness as participants must be aware that they are experiencing TUT before a response can be made. Therefore, participants may have underreported instances of TUT due to the added requirement. However, the advantage of self-caught measures over probe-caught measures is that participants are able to report multiple TUT events within one probe period. Future research should examine if the same results are observed with probe-caught measures.

Lastly, this study was conducted online due to the COVID-19 pandemic. Most studies in the field using the metronome response task, are performed in-person. Hence, it is possible that variation in computers and input devices used in the study could have inflated noise in the data. In fact, this could be one explanation as to why the H values we observed were lower than those typically observed in repetitive human actions.

## Conclusion

In summary, the present experiment investigated the relationship between tapping structure variability and task-unrelated thought. In doing so, there were several notable trends. First, the metronome response task produced inter-beat interval patterns similar to patterns observed in the study of gait and other intentional behaviors [24,44,64,70,74,78,81,89]. Second, TUT generally increased with decreases in the temporal structure of tapping sequences. This finding was not universal, however, as this trend was not observed when tapping was paced by a *Periodic* metronome, though the difference in the relationship compared to the other metronome conditions was not significant. That last point has important consequences for the generality of the metronome response task when used with a *Periodic* metronome given that the structure of variability and the rate of TUT may not be representative of everyday life. In addition, we note that there seems to be a strong correspondence between variability (both magnitude and structure) observed in the presence of TUT and variability observed in human movement science, more generally. As such, the current results suggest a number of research ideas related to TUT as an explanatory device understanding variability in critical processes such as walking and running.
